# Extended-release buprenorphine induction in opioid non-tolerant incarcerated individuals

**DOI:** 10.1016/j.dadr.2024.100261

**Published:** 2024-07-20

**Authors:** Michael S. Gordon, Thomas R. Blue, Frank J. Vocci, Shannon G. Mitchell, Kevin R. Wenzel, Marc Fishman

**Affiliations:** a Friends Research Institute, Inc., 1040 Park Avenue, Suite 103, Baltimore, MD 21201, United States; b Maryland Treatment Centers, 3800 Frederick Ave, Baltimore, MD 21229, United States

**Keywords:** Jail, Sublingual buprenorphine, Extended-release injectable buprenorphine

## Abstract

**Background:**

Buprenorphine maintenance treatment remains unavailable in most jails in the US. We provide data on a four-day rapid sublingual buprenorphine (SL-B) induction strategy followed by a weekly dose of extended-release injectable buprenorphine (XR-B) with incarcerated individuals with opioid use disorder (OUD) who were not opioid tolerant.

**Methods:**

Between October 2020 to April 2024, *N* = 65 individuals with an opioid use disorder in jails participating in a larger randomized, controlled trial received SL-B and XR-B prior to release. Primary outcomes included completing the proposed dose induction and any reported adverse events (AEs).

**Results:**

Sixty-five individuals received SL-B dose induction from our team’s medical staff, 53 (81.5 %) completed the four-day SL-B dose induction and received their first weekly XR‑B injection on day 5. Of the 65 individuals, 10 (15.38 %) participants reported AEs during the dosing period and/or in the week following the dosing period. All but one of the AEs were rated as mild. One participant experienced a serious adverse event in the week following dose induction. The study medical team determined that this was unlikely to be related to the intervention.

**Discussion:**

Overall, our study findings demonstrate the feasibility of implementing a four-day sublingual dose induction followed by a weekly XR-B injection with incarcerated individuals who are not opioid tolerant. This study provides important data to illustrate a dose induction strategy that might assist in reducing illicit diversion in jails, which is a main barrier to buprenorphine delivery cited by correctional administrators.

## Highlights

• Buprenorphine treatment remains unavailable in most jails in the US.
• Limited guidelines exist on the clinical dosing of buprenorphine for incarcerated individuals.
• Four-day sublingual dose induction followed by an injection of a weekly dose of extended-release buprenorphine with incarcerated individual is feasible.

## 1 Introduction

At year-end 2021, approximately 1.7 million individuals were incarcerated in state or federal prisons or local jails in the United States (Carson & Kluckow, 2023). Incarcerated individuals face disproportionately high rates of opioid use disorder (OUD) compared to non-incarcerated individuals, and are at an elevated risk of opioid relapse, overdose death, and recidivism after release (Binswanger et al., 2020, Keen et al., 2020). Furthermore, very few incarcerated individuals receive adequate treatment (Brinkley-Rubinstein et al., 2018; Farahmand, Modesto-Lowe, Chaplin, 2017; Krawczyk, Picher, Feder & Saloner, 2017), even though existing data indicates medications for OUD (MOUD) can improve community outcomes (Evans et al., 2022; Mace et al., 2020; Malta et al., 2019). For example, providing jail-initiated buprenorphine to individuals with an OUD has been shown to reduce recidivism (Evans et al., 2022) and overdose death after release (Pourtaher et al., 2024). There is limited national data on the number of jails in the US that offer MOUD. It’s estimated that less than 14 % of jails in the US provide buprenorphine and/or methadone prior to release to the community (Thakrar et al., 2021). Buprenorphine treatment still remains unavailable in most jails and prisons in the US (Grella et al., 2020; Macmadu et al., 2020), largely due to concerns over diversion (Bandara et al., 2021; Doernberg et al., 2019).

BRIXADI™ is a depot subcutaneous extended-release buprenorphine (XR-B) injection that was approved for treatment of moderate to severe OUD by the Food and Drug Administration (FDA) in May 2023. It provides sustained buprenorphine release in both weekly (8 mg, 16 mg, 24 mg, 32 mg) and monthly (64 mg, 96 mg, 128 mg) formulations. BRIXADI™ has been evaluated in several Phase 1–3 clinical trials (Albayaty et al., 2017; Haasen, Linden, & Tiberg, 2017; Walsh et al., 2017; Lofwall et al., 2018) and its safety has been consistent with sublingual buprenorphine, except for mild/moderate injection-site adverse events (Lofwall et al., 2018). XR-B administration promotes delivery and medication adherence, while potentially minimizing risks of diversion, misuse, and accidental exposure. Furthermore, it has the potential to reduce opioid relapse and overdose deaths in the high-risk period immediately after community reentry.

This study provides data to support the implementation of a four-day SL-B dose induction followed by the first weekly dose of XR-B among opioid non-tolerant individuals with OUD in a carceral setting (jail as opposed to prison). The protocol for SL-B induction was 2 mg for 2 days (Monday, Tuesday), 4 mg for 2 days (Wednesday, Thursday), and an 8 mg weekly dose of XR-B (reported equivalence to ≤ 6 mg per package insert on day 5 (Friday).

Our primary outcome was the proportion of participants successfully completing four-days of SL-B dose induction and receiving the first weekly XR-B dose on day five (yes *vs*. no). Our secondary outcome was adverse events reported during the rapid SL-B dose induction and adverse events reported within one week of dose induction (yes *vs*. no).

## 2 Materials and methods

The primary study is a multi-site, randomized, controlled trial of XR-B (BRIXADI™) versus extended-release naltrexone (XR-NTX; VIVITROL®) in Maryland jails. A five-site, open-label, equivalence design randomly assigned 240 adults with a history of OUD, who were nearing release, to one of two treatment arms: XR-B in jail, followed by 6 monthly injections post-release at a community treatment program; or XR-NTX in jail followed by 6 monthly injections post-release at a community treatment program (see Gordon et al., 2021 for additional information on methods).

This secondary analysis involved a subset of participants from the primary study. At three of the five participating county jails, the research study medical team (as opposed to the jail medical provider) was required to provide all sublingual buprenorphine (SL-B) dosing prior to the administration of the first weekly injection of XR-B. The jails were located in rural and suburban areas of Maryland. At these three jail sites, our study protocol was to provide four days of sublingual buprenorphine followed by a first XR-B weekly injection on day five. The other two sites where the jail medical provider administered dosing typically took longer than four days of SL-B dosing to induct the participants. However, the jail medical providers were asked to follow the same initial dose induction of 2 mg, 2 mg, 4 mg, 4 mg. After the initial dose induction, participants continued with multiple days of 8 mg SL-B dosing prior to receiving their first weekly XR-B injection by our study medical team.

### 2.1 Selection and recruitment of patients

Incarcerated individuals scheduled to be released within 120 days, who met DSM-5 criteria of OUD, and were willing to be randomized to receive either XR-B or XR-NTX were eligible for participation. Participants randomized to receive XR-B at the three county jails where the study medical team was responsible for SL-B dosing are included in the present study. All individuals underwent a medical evaluation by the study team to determine general health. We excluded individuals with medical conditions or psychiatric illnesses judged too unstable to allow participation.

Incarcerated individuals who were actively experiencing opioid withdrawal were not eligible. Research assistants (RAs) worked with jail staff at the three county jails to identify individuals interested in participating. Individuals were either referred by the jail medical team, addiction counselor, case management, or responded to study flyers posted throughout the facility. In addition, incarcerated individuals could send direct messages to our research team through a jail-based internet kiosk system. All individuals were pre-screened by research staff for DSM-5 criteria. After individuals signed the informed consent form, they had to pass an informed consent quiz. After passing the initial screening and signing the informed consent form, individuals met with the study medical team for a 45–60-minute health assessment to further determine eligibility. All individuals were non-tolerant for opioids prior to induction as evidenced by recent drug taking history and confirmed by a negative urine drug screen. Participants were screened for the following substances: opiates (200 ng/ml), methadone (300 ng/ml), buprenorphine (10 ng/ml), morphine (300 ng/ml), fentanyl (20 ng/ml), and oxycodone (100 ng/ml). The protocol for SL-B induction was 2 mg for 2 days (Monday, Tuesday), 4 mg for 2 days (Wednesday, Thursday), and an 8 mg weekly dose of XR-B (reported equivalence to ≤ 6 mg per package insert on day 5 (Friday). However, the dosing protocol allowed for flexibility based on participant dose induction adverse event reporting, allowing our clinicians to extend the period of SL-B dose induction > four days, if needed, on a case-by-case basis to ensure safety and tolerability. According to BRIXADI™ dosing information (https://www.brixadi.com/pdfs/brixadi-prescribing-information.pdf) the corresponding dose of < 6 mg daily dose of transmucosal buprenorphine is 8 mg BRIXADI™ (weekly). Subsequent XR-B doses included 16 mg weekly dose, 24 mg weekly dose and a final target goal of 128 mg monthly dose prior to release from jail. Participants receiving the 24 mg weekly dose could then be transferred to the 128 mg monthly dose before release. Inductions were preferably started on Monday to avoid weekend dosing due to staff constraints.

The study was approved by Western Institutional Review Board and the US Office of Human Research Protections. The study is registered at ClinicalTrials.gov (NCT04408313) and is being monitored by a NIDA Data and Safety Monitoring Board. The study team adhered to all Declaration of Helsinki principles.

### 2.2 Buprenorphine exposure and adverse events

The study timeline began with the participant’s first daily dose of SL-B (Day 1) and ended with the initial XR-B dose of 8 mg (Day 5). Our primary outcome was proportion of participants successfully completing four days of SL-B dose induction and receiving the first weekly XR-B dose on day five (yes *vs*. no). Our secondary outcome was adverse events reported during the rapid SL-B dose induction and adverse events reported within one week of dose induction (yes *vs*. no). We also report types of adverse events.

### 2.3 Statistical analyses

Descriptive statistics were conducted for baseline demographic characteristics and for the primary outcomes, completing dose induction (yes vs. no) and any adverse events (yes vs. no).

## 3 Results

### 3.1 Baseline demographic characteristics

Between October 2020 to April 2024, 65 individuals were provided SL-B dosing by our study medical team. Of the 65 individuals, a plurality self-identified as White (*n* = 36, 55.38 %), the second most common self-identified race was Black/African American (*n* = 26, 40.00 %); four individuals self-identified as Hispanic/Latino (6.15 %). The majority were male (*n* = 51, 78.46 %) and the mean age was 33.55 years old (*SD* = 8.70) at randomization. Participants had been incarcerated in jail for the past 183.78 days on average (*M*dn  = 140, *SD* = 161.11) at baseline. In the 30 days prior to their index incarceration, 34 (52.31 %) participants reported using heroin on an average of 21.32 days (*M*dn  = 30, *SD* = 11.29), 18 (27.69 %) reported using fentanyl on an average of 24.06 days (*M*dn  = 30, *SD* = 9.31), and 31 (47.69 %) reported using other opioids (e.g., oxycodone, codeine) on an average of 20.39 days (*M*dn  = 29, *SD* = 11.13). Thirty-eight (58.46 %) participants reported having been prescribed an MOUD in the past; 26 (40.00 %) individuals reported an average of 16.85 lifetime months of buprenorphine treatment (*M*dn  = 12, *SD* = 18.84).

### 3.2 Buprenorphine exposure and adverse events

Of the sixty-five individuals who received SL-B dose induction from our team’s medical staff, 53 (81.54 %) completed the proposed four-day SL-B dose induction prior to receiving their first weekly XR-B injection and therefore met the primary outcome. Of the 53 individuals that completed the proposed dose induction, 13 completed the dose induction but deviated slightly from the dosing protocol. Seven individuals received SL-B dosing on three consecutive days (*n* = 5 received doses of 2, 2, 4; *n* = 2, received doses of 2, 4, 4) prior to their first weekly XR-B injection of 8 mg. Reasons for 3 day dosing in these five individuals were: one was transferred to another jail; two were randomized on a Monday and dosing began on Tuesday; and two were due to a holiday week. Four individuals received three doses over four days (*n* = 2 received doses of 0, 2, 2, 4; *n* = 1 received 2, 0, 2, 2; *n* = 1 received 2, 2, 0, 4) with two being in court for a full day; one refusing to get out of bed; and one was due to a holiday week. Two individuals completed the protocol dose induction of SL-B but were administered a 16 mg weekly XR-B dose on day five as opposed to 8 mg due to a medication error. Even though these 13 individuals deviated from the dosing protocol they still successfully received their first weekly XR-B injection within the five-day protocol with no adverse events and were included in the primary outcome.

Twelve individuals (18.5 %) did not meet the primary outcome. Eight of the 12 individuals required > 5 days of SL-B dose induction prior to receiving their first weekly XR-B dose for various logistical reasons. Three individuals were unexpectedly released from jail to a community residential program during their jail induction week. Our research staff had to meet with community treatment providers to explain the study and obtain approval to continue individuals at the respective programs, which delayed the first XR-B injection (individuals received 15, 9, and 8 days of SL-B induction prior to their first injection). Two individuals were dosed > 5 days due to scheduling conflicts with our medical staff and jail (individuals received 6, and 7 days of SL-B induction prior to their first injection). Two individuals were dosed > 5 days due to COVID restrictions (both received 7 days induction prior to first weekly injection). One individual was caught diverting non-study drug buprenorphine and was moved to a different county jail and we had to meet with jail administrators to discuss the study operations (this individual received 5 days of SL-B prior to first weekly injection). Four individuals discontinued SL-B dosing during the first week of induction. One participant completed five days of SL-B dose induction before refusing the weekly XR-B injection due to a preference for Vivitrol. One participant completed three days of dose induction and discontinued it due to a preference for Vivitrol and adverse events (nausea, vomiting, and diarrhea). One participant received one day of SL-B and then discontinued due to receiving a detainer in a nearby county that was not offering any MOUDs at the time. The participant was afraid of having to detoxify from buprenorphine. One participant received three days of SL-B before reporting they were afraid of needles and had a preference to stay on SL-B and did not want to switch to the injectable formulation. Table 1, Table 2

**Table 1:** Dose induction completion.

Achieved (*N* = 53, 81.54 %)	completed protocol as written (*n* = 40)
	completed protocol with minor modifications (*n* = 13)
	received SL-B dosing on three consecutive days (*n* = 7)
	received three doses over four days (*n* = 4)
	completed the dosing but received 16 mg weekly on day 5 (*n* = 2)
Not Achieved (*N* = 12, 18.46 %)	released to a residential facility (*n* = 3)
	scheduling conflicts at the jail medical unit (*n* = 2)
	COVID restrictions (*n* = 2)
	caught diverting (*n* = 1)
	preference for Vivitrol (*n* = 1) preference for Vivitrol and adverse events (*n* = 1)
	detainer in another jail that was not offering MOUD (*n* = 1)
	afraid of needles and had a preference to continue SL-B (*n* = 1)

**Table 2:** Adverse events.

Adverse events, *N* = 19^a^	Related to study drug	Severity
Sedation/drowsiness, *n* = 4	Probably	Mild
Nausea, *n* = 3	Probably	Mild
Vomiting, *n* = 3	Probably	Mild
Swelling/Edema, *n* = 3	Probably	Mild
Constipation, *n* = 2	Probably	Mild
Diarrhea, *n* = 1	Probably	Mild
Headache, *n* = 1	Probably	Mild
Insomnia, *n* = 1	Probably	Mild
Sickle cell crisis, *n* = 1	Remotely	Severe

a *N* = 10 unique individuals reported 19 adverse events.

Of the 65 individuals who received SL-B dosing, 10 (15.38 %) reported adverse events (AEs) during the dosing period and/or in the week following the dosing period. Six participants (9.23 %) reported 12 AEs that occurred during the dosing period and 6 participants (9.23 %) reported 7 AEs that occurred in the week following the dosing period. Of these 19 AEs, the most frequent were sedation/drowsiness (*n* = 4, 21.05 %), nausea (*n* = 3, 15.79 %), vomiting (*n* = 3, 15.79 %) and swelling (n = 3, 15,79 %). All but one of the AEs were rated as mild. One participant experienced a serious adverse event in the week following dose induction. The participant was hospitalized for three nights for an urgent transfusion due to a sickle cell crisis. The participant was also treated for presumptive pneumonia. The participant continued to receive monthly XR-B after being discharged from the hospital. The study medical team reviewed the participant’s medical records and determined that this serious adverse event was not related to the study drug/procedures.

## 4 Discussion

To our knowledge this is the first study in the US to implement a four-day SL-B dose induction followed by a day five XR-B weekly injection with incarcerated individuals who were not opioid tolerant. There is limited literature providing guidelines on the clinical dosing of buprenorphine/naloxone for incarcerated individuals who have lost their tolerance to opioids (Regenstreif et al., 2022). The very few studies conducted in the US initiated SL-B with incarcerated individuals who were not opioid tolerant, starting at 1 mg or 2 mg and continuing for several weeks prior to release (Garcia et al., 2007; Gordon 2014; Springer et al., 2010). Overall, our study findings demonstrate the feasibility of implementing a four-day sublingual dose induction followed by a weekly XR-B injection with incarcerated individuals who are not opioid tolerant. Importantly, 53 of the 65 participants (81.54 %) who initiated SL-B while incarcerated were successfully inducted onto XR-B. Moreover, if the 8 individuals who were inducted > 5 days, for various logistical reasons, to begin initiation of XR-B are included, the rate would be 93.8 % successfully initiated XR-B within 15 days. Twelve of the 65 participants (18.46 %) did not meet the primary outcome. The primary barriers were logistical (i.e., scheduling, staffing, early release) rather than clinical (i.e., difficulty tolerating the titration, AEs). This paper provides evidence for the clinical acceptability of the induction protocol described.

Overall, our findings contribute to the limited guidance available, and expand our knowledge base to XR-B. This is especially important because many correctional administrators prioritize the importance of reducing illicit SL-B diversion and XR-B may therefore be a particularly attractive option. Additionally, shorter duration induction may reduce the chances of not receiving a first dose of XR-B in jail due to changes in motivation or logistical barriers. Our findings are also timely as carceral settings are increasingly adopting MOUD into usual care, although overall rates of MOUD availability remain low.

Despite these findings, there are several limitations that should be noted. First the small sample size limits generalizability. Second, due to the variations in dose induction procedures at the other study sites, we do not have a proper control group to compare to the 63 participants in this study. Third, we did not gather qualitative data on the acceptability of the dosing procedure. Participants met with nursing staff daily during SL-B induction to discuss any AEs or difficulties tolerating the titration, but it is possible that some participants withheld information on discomfort because MOUD was generally not available outside of the study. Despite the limitations, there were only 12 AEs that occurred during the SL-B dosing period and seven that were reported within one week of the SL-B dosing period. We should note that patient preference, adverse event monitoring, including paying close attention to precipitated withdrawal, nausea, and oversedation should be of the utmost importance during the induction period (first week). Given the risk of fentanyl overdose, and high rate of turnover (release from jail), it is important to consider a rapid SL-B and XR-B initiation with this high-risk vulnerable population. Furthermore, it should be highlighted that there has been an increased nationwide focus on implementing MOUDs in carceral settings. In fact, recently the Department of Justice (2022) issued specific guidance on protections for individuals with OUD under the American with Disabilities Act which might increase buprenorphine in carceral settings.

## Role of funding source

This study was supported by the 10.13039/100000002National Institutes of Health/ 10.13039/100000026National Institute on Drug Abuse (Grant#UG1DA050077). Braeburn provided in-kind study drug (extended, release buprenorphine BRIXADI).

## CRediT authorship contribution statement

**Kevin J Wenzel:** Writing – review & editing. **Marc Fishman:** Writing – review & editing, Investigation. **Shannon G Mitchell:** Writing – review & editing, Investigation. **Thoma R Blue:** Writing – review & editing, Writing – original draft, Validation, Project administration, Methodology, Investigation, Formal analysis, Data curation, Conceptualization. **Frank J Vocci:** Writing – review & editing, Methodology, Investigation, Funding acquisition, Conceptualization. **Michael Scott Gordon:** Writing – review & editing, Writing – original draft, Validation, Supervision, Resources, Project administration, Methodology, Investigation, Funding acquisition, Formal analysis, Data curation, Conceptualization.

## Declaration of Competing Interest

MG, FV, and TB receive in-kind study drug from Braeburn and Alkermes. FV has received meals from Braeburn. SM receives in-kind study drug from Braeburn. MF has been a consultant for Indivior, Alkermes, Drug Delivery LLC, has been the recipient of research funding from Alkermes and Indivior, and has received study drug from Alkermes, Indivior and Braeburn.
